# Assessment of genetic diversity in *Vigna unguiculata* L*.* (Walp) accessions using inter-simple sequence repeat (ISSR) and start codon targeted (SCoT) polymorphic markers

**DOI:** 10.1186/s12863-017-0567-6

**Published:** 2017-11-17

**Authors:** David Okeh Igwe, Celestine Azubike Afiukwa, Benjamin Ewa Ubi, Kenneth Idika Ogbu, Omena Bernard Ojuederie, George Nkem Ude

**Affiliations:** 1 Department of Biotechnology, Ebonyi State University, Abakaliki, Ebonyi State P.M.B 053 Nigeria; 2 Biotechnology and Research Development Centre, Ebonyi State University, Abakaliki, Ebonyi State Nigeria; 3 Department of Biological Sciences, College of Natural and Applied Sciences, Bells University of Technology, Ota, Ogun State Nigeria; 4 Department of Natural Sciences, Bowie State University, 14000 Jericho Park Road, Bowie, USA

**Keywords:** *Vigna unguiculata*, ISSR markers, SCoT markers, Genetic diversity, Shannon’s information index, Polymorphism

## Abstract

**Background:**

Assessment of genetic diversity of *Vigna unguiculata* (L.) Walp (cowpea) accessions using informative molecular markers is imperative for their genetic improvement and conservation. Use of efficacious molecular markers to obtain the required knowledge of the genetic diversity within the local and regional germplasm collections can enhance the overall effectiveness of cowpea improvement programs, hence, the comparative assessment of Inter-simple sequence repeat (ISSR) and Start codon targeted (SCoT) markers in genetic diversity of *V. unguiculata* accessions from different regions in Nigeria. Comparative analysis of the genetic diversity of eighteen accessions from different locations in Nigeria was investigated using ISSR and SCoT markers. DNA extraction was done using Zymogen Kit according to its manufacturer’s instructions followed by amplifications with ISSR and SCoT and agarose gel electrophoresis. The reproducible bands were scored for analyses of dendrograms, principal component analysis, genetic diversity, allele frequency, polymorphic information content, and population structure.

**Results:**

Both ISSR and SCoT markers resolved the accessions into five major clusters based on dendrogram and principal component analyses. Alleles of 32 and 52 were obtained with ISSR and SCoT, respectively. Numbers of alleles, gene diversity and polymorphic information content detected with ISSR were 9.4000, 0.7358 and 0.7192, while SCoT yielded 11.1667, 0.8158 and 0.8009, respectively. Polymorphic loci were 70 and 80 in ISSR and SCoT, respectively. Both markers produced high polymorphism (94.44–100%). The ranges of effective number of alleles (Ne) were 1.2887 ± 0.1797–1.7831 ± 0.2944 and 1.7416 ± 0.0776–1.9181 ± 0.2426 in ISSR and SCoT, respectively. The Nei’s genetic diversity (H) ranged from 0.2112 ± 0.0600–0.4335 ± 0.1371 and 0.4111 ± 0.0226–0.4778 ± 0.1168 in ISSR and SCoT, respectively. Shannon’s information index (I) from ISSR and SCoT were 0.3583 ± 0.0639–0.6237 ± 0.1759 and 0.5911 ± 0.0233–0.6706 ± 0.1604. Total gene diversity (Ht), gene diversity within population (Hs), coefficient of gene differentiation (Gst) and level of gene flow (Nm) revealed by ISSR were 0.4498, 0.3203, 0.2878 and 1.2371 respectively, while SCoT had 0.4808, 0.4522, 0.0594 and 7.9245.

**Conclusions:**

Both markers showed highest genetic diversity in accessions from Ebonyi. Our study demonstrated that SCoT markers were more efficient than ISSR for genetic diversity studies in *V. unguiculata* and can be integrated in the exploration of their genetic diversity for improvement and germplasm utilization.

**Electronic supplementary material:**

The online version of this article (10.1186/s12863-017-0567-6) contains supplementary material, which is available to authorized users.

## Background

*Vigna unguiculata* (L.) Walp (cowpea, 2n = 2× = 22) is a dicotyledonous annual crop in the family Fabaceae and it is commercially cultivated in both tropical and sub-tropical parts of the world [1–3]. It provides 53% carbohydrate, 2% fat and 22 to 33% protein, including a high content of lysine, an essential amino acid for human metabolism [4–7]. It is used as a ground cover crop for erosion control, weed suppressor due to its allopathic constituents, and as animal feed [8]. About 10% of the grainy-crop is grown as green leafy vegetable and fodder in Africa [9]. It fixes 80% nitrogen for its growth demand from the atmosphere [10–12]. This quality of nitrogen fixation contributes to sustainability of cropping systems and improvement of soil fertility in marginal lands (11–12]. The global production of cowpea was estimated to be 2.27 million tonnes, out of which Nigeria accounted for 850, 000 t [13, 14]. Nigeria, Burkina Faso, Niger, Brazil, Haiti, India, Sri Lanka, Australia and the United States are the principal producers and account for over 70% in the production of the crop [15–17]. Nigeria has been reported to be the second greatest consumer of cowpea in the world [18]. It is widely cultivated due to its palatability and low cost of production [19, 20]. The crop adapts to sandy soils and warm conditions but very sensitive to low temperatures [16, 21]. It is an essential constituent of cropping systems of the drier tropics such as Asia, the Middle East, Southern Europe, Africa, Southern USA, and Central and South America [22]. The crop has relatively a feature of drought tolerance and this enhances its cultivation in the savanna and forest savanna zones of West Africa [6, 23, 24]. Cowpea management requires low resource inputs compared to other staple crops and is affordable to poor farmers [23]. Factors including drought stress, high salinity and changes in temperature can negatively affect production yield [25, 26]. Effects of these factors on the crop can be minimized through effective breeding programme with unique accessions identifiable and selectable using informative molecular markers. Cowpea is known to be self-pollinating and the presence of diverse accessions is imperative to improve its low genetic base which is narrow [27–29]. However, identification of individual accession into different groups is mostly done based on the morphological features that do not necessarily demonstrate the real genetic relatedness and often affected by environmental factors hence, the need for use of molecular markers. Molecular markers such as Randomly amplified polymorphic DNA (RAPD), Simple sequence repeat (SSR), Amplified fragment length polymorphism (AFLP) and Inter-simple sequence repeat (ISSR) have been applied in the estimation of genetic diversity, genetic relationship and germplasm management and conservation in *V. unguiculata* [29–31]. Knowledge of the genetic diversity available within the local and regional germplasm collections can enhance the overall effectiveness of cowpea improvement programs [32]. Use of SCoT markers that were developed to target the conserved regions of genome across various plant species due to their longer primer lengths and high annealing temperatures would be more efficient and reliable compared to other arbitrary markers [33]. Inter-simple sequence repeat markers are arbitrary markers that target multiple genomic loci and amplify DNA segments present between two identical microsatellite regions that are opposite with each other in orientation [34]. Apparently, to the best of our knowledge, comparative assessment of ISSR and SCoT markers has not been applied in *V. unguiculata* for its genetic diversity assessment. The objective of this study was to compare the efficacies of ISSR and SCoT polymorphic markers to assess the genetic diversity in accessions of *V. unguiculata* from different regions in Nigeria.

## Methods

### Sample collection and DNA extraction

Eighteen cowpea accessions were collected from different local government areas in Abia, Enugu and Ebonyi States including three samples from International Institute of Tropical Agriculture, (IITA), Ibadan, Nigeria (Table 1). Approximately 100 mg of fresh young leaves of *V. unguiculata* were collected from the screenhouse for DNA extraction using ZYMOGEN KIT (Zymo Research Corporation, USA), according to its manufacturer’s instructions.

**Table 1:** List of cowpea accessions used for comparative genetic diversity study and their sources

Location	L.G.A	State	Accession
Amuda	Umunneochi	Abia	AbCp-1
Eluama	Isiukwuato	Abia	AbCp-2
Lokpaukwu	Umunneochi	Abia	AbCp-3
Mbawusi	Isiala Ngwa North	Abia	AbCp-4
Abakpa	Nike	Enugu	AbCp-5
Nsuka	Enugu	Enugu	EnCp-1
Udi	Enugu	Enugu	EnCp-2
Eziagu	Enugu	Enugu	EnCp-3
Agbani	Enugu	Enugu	EnCp-4
Akwuke	Enugu	Enugu	EnCp-5
Okwerike	Ikwo	Ebonyi	EbCp-1
Umuezeokoha	Ezza North	Ebonyi	EbCp-2
Effium	Ezzamgbo	Ebonyi	EbCp-3
Akpugo	Nkanu South	Ebonyi	EbCp-4
Liberty	Nike	Ebonyi	EbCp-5
Ibadan	Akinyele	Oyo	TVu–264
Ibadan	Akinyele	Oyo	Ife Brown
Ibadan	Akinyele	Oyo	IT84S-2246

*AbCp* Abia cowpea, *EnCp* Enugu cowpea, *EbCp* Ebonyi cowpea, *L.G.A* Local government area

### Polymerase chain reaction and agarose gel electrophoresis

Polymerase chain reaction (PCR) amplification was performed in volume of 25 μL which consisted of 2.0 μL 100 ng DNA, 2.5 μl of 10× Taq Buffer (BIOLINE, Massachusetts, USA), 1.5 μl of 50 mM MgCl_2_ (BIOLINE, Massachusetts, USA), 2.0 μl of 2.5 mM dNTPs (BIOLINE, Massachusetts, USA), and 0.2 μl 500 U *Taq* DNA polymerase (BIOLINE, Massachusetts, USA), 1.0 μl of 10 μM each of SCoT or ISSR primer pair and 15.05 μl of 500 ml diethyl pyrocarbonate (DEPC)-treated water (INVITROGEN, Carlsbad, CA, USA). The list of ISSR and SCoT markers, their sequences, GC content and annealing temperatures are presented in Tables 2. The PCR cycling profile used for the reaction consisted of an initial step at 94 °C for 5 min., followed by 35 cycles of 94 °C for 30 s, 72 °C for 1 min, and a 10 min final extension at 72 °C. Eight (8) μl of the PCR reaction products were electrophoresed in a 1.5% agarose gel containing 0.5 mg/ml ethidium bromide and photographed on Transilluminator UV light (Fotodyne Incorporated, Analyst Express, USA).

**Table 2:** List of primers, their sequences, percentage GC contents and annealing temperatures

Primer name	Primer sequence (5′-3′)	% GC	Annealing temperature
ISSR825	ACACACACACACACT	47	55
UBC835	AGAGAGAGAGAGAGAGYC	56	60
UBC814	CTCTCTCTCTCTCTCAT	47	55
UBC826	ACACACACACACACACC	53	57
UBC827	ACACACACACACACACG	53	57
UBC840	GAGAGAGAGAGAGAGATT	44	55
UBC808	AGAGAGAGAGAGAGAGC	53	57
UBC811	GAGAGAGAGAGAGAGC	53	57
UBC868	GAAGAAGAAGAAGAAGAA	33	51
UBC901	CACACACACACACACARY	44	55
SCoT2	CAACAATGGCTACCACCC	55	48
SCoT13	ACGACATGGCGACCATCG	61	48
SCoT16	ACCATGGCTACCACCGAC	61	48
SCoT20	ACCATGGCTACCACCGCG	66	48
SCoT22	AACCATGGCTACCACCAC	55	48
SCoT24	CACCATGGCTACCACCAT	50	48
SCoT28	CCATGGCTACCACCGCCA	66	48
SCoT33	CCATGGCTACCACCGCAG	61	50
SCoT35	CATGGCTACCACCGGCCC	72	48
SCoT36	GCAACAATGGCTACCACC	55	48

### Data analyses

Data matrices of Inter-simple sequence repeat and start codon targeted polymorphic marker profiles were generated by scoring (1) for presence and (0) for absence of individual allele. The obtained data matrices were used for phylogenetic reconstruction using Unweighted Pair Group Mean with Arithmetic (UPGMA) and dissimilarity index in Jaccard’s option [35] were used for the analysis. The analysis was conducted using NTSYSpc software version 2.02. Furthermore, the genetic diversity, allele frequency and polymorphic information content (PIC) were computed using PowerMarker (Version 3.25). Genetic diversity and population structure analyses of the accessions were analysed using POPGENE software version 1.32.

## Results

### Genetic diversity revealed by inter-simple sequence repeat (ISSR) markers

**Fig. 1:**
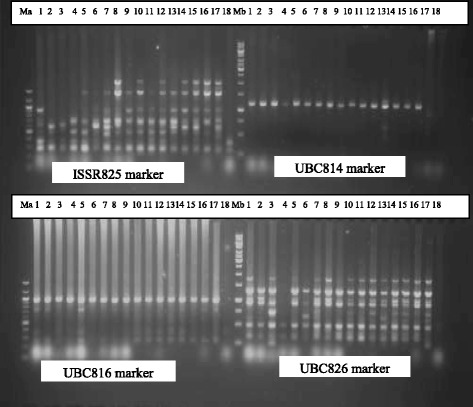
Amplification profiles of eighteen cowpea DNA samples using four ISSR markers: Ma = 100 bp step DNA ladder and Mb = 1 kb DNA ladder, 1–5 (AbCp-Abia cowpea), 6–10 (EnCp–Enugu cowpea), 11–15 (EbCp–Ebonyi cowpea), 16–18 (IITA, Ibadan, Oyo) and L.G.A = Local government area

**Fig. 2:**
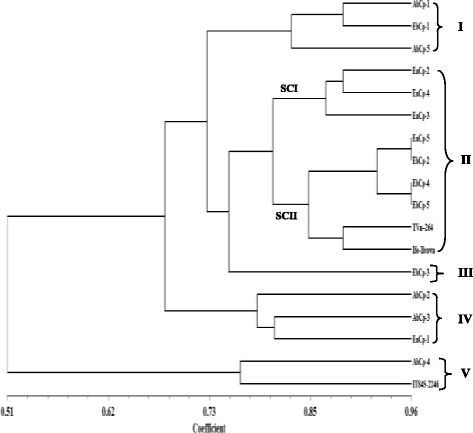
Dendrogram of 18 *Vigna unguiculata* amplified with Inter-simple sequence repeat markers

**Fig. 3:**
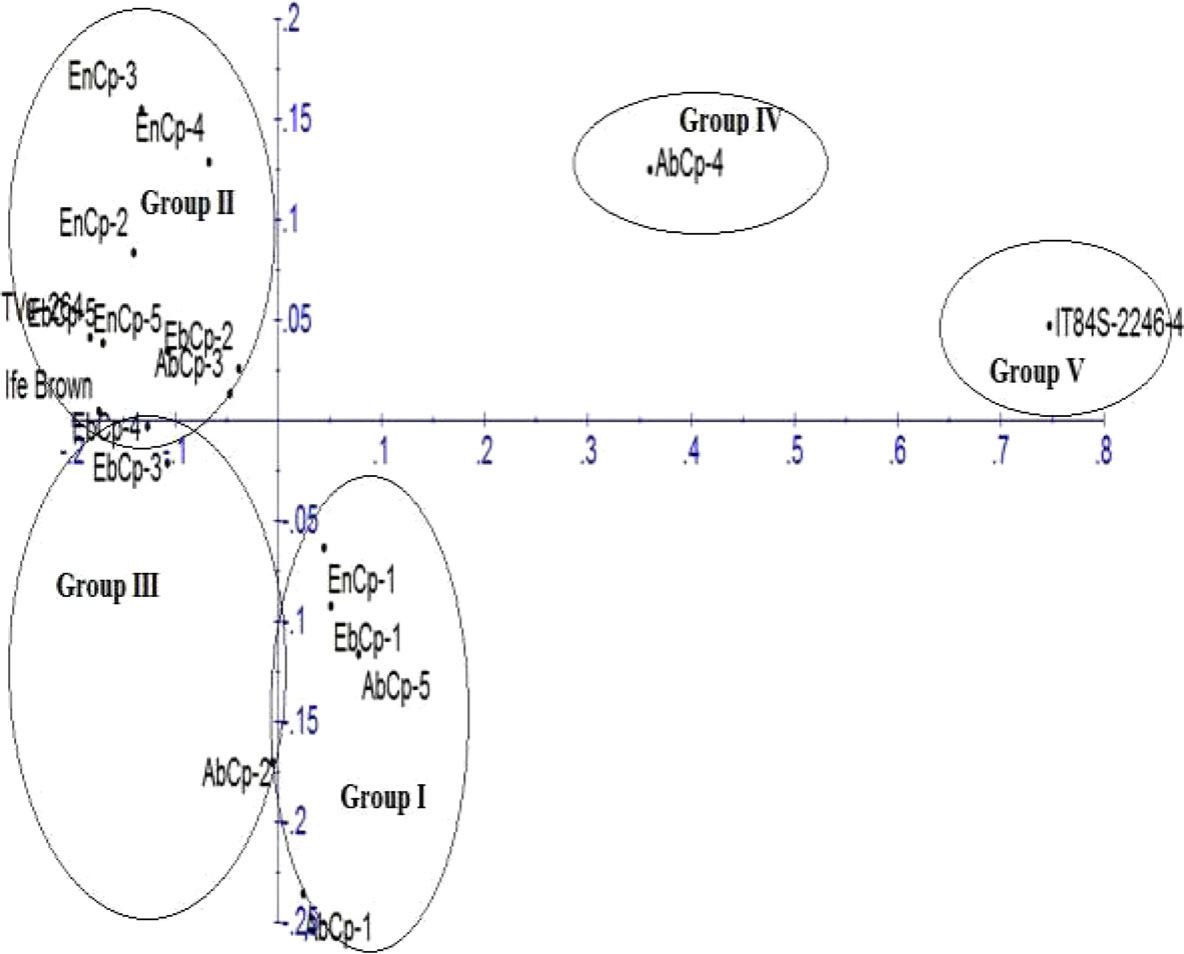
Principal component analysis of 18 *Vigna unguiculata* based on Inter-simple sequence repeat markers

To access the level of genetic diversity in 18 accessions of *V. unguiculata*, a total of 10 ISSR primers were tested. Four (4) out of the 10 primers produced scorable bands that were used in the diversity analysis (Fig. 1). A dendrogram of the 18 accessions generated from UPGMA procedure, grouped the accessions into five major clusters at similarity index of 0.751 (Fig. 2). Cluster I consisted of AbCp-1, EbCp-1, and AbCp-5 consisting of accessions from Abia (AbCp), Ebonyi (EbCp) and Enugu (EnCp) States of Nigeria. Cluster II was subdivided into two subclusters, SCI and SCII. SCI had three accessions EnCp-2, EnCp-4 with EnCp-3 all from Enugu State. SCII contained accessions from different locations but showed that accessions EbCp-2 and EnCp-5 from Enugu were more closely related as well as accessions EbCp-4 and EbCp-5 from Ebonyi while accessions TVu-264 and Ife Brown from IITA were closer together. The accessions contained in this cluster were collected from Enugu State, Ebonyi State and International Institute of Tropical Agriculture (IITA), Ibadan, Oyo State. Cluster III contained only an accession EbCp-3 from Ebonyi State. Cluster IV contained accessions AbCp-2, AbCp-3 (Abia State) and EnCp-1(Enugu State) which was genetically isolated from the other accessions. Cluster V grouped accession AbCp-4 from Abia State together with IT84S-2246 from IITA. Principal component analysis of the generated amplicons also resulted in five groups (Fig. 3) corresponding to the numbers of clusters obtained from the dendrogram. Each group was a representative of unique accession.

The four (4) ISSR primers amplified a total of 32 alleles. The amplified alleles from each primer ranged from 4 to 14, with a mean of 9.4000 (Table 3). Polymorphic information content (PIC) values ranged from 0.2834–0.9073 with a mean value of 0.7192. The ISSR markers ISSR825, UBC816 and UBC826 were found to be polymorphic while UBC814 was monomorphic with the least PIC value. The genetic diversity values ranged from 0.2963–0.9136 with a mean of 0.7358 while major allele frequency spanned from 0.1667–0.8333 with a mean value of 0.3778. Allelic scores, counts and frequencies detected from the accessions of *V. unguiculata* using ISSR markers were recorded (Additional file 1: Table S1). The genetic diversity in Ebonyi (EbCp) population was identified to be the highest, with effective number of alleles (Ne), Nei’s gene diversity (H) and Shannon’s Information index (I) values of 1.9831, 0.4957 and 0.6888, respectively (Table 4 **).** Contrarily, the genetic diversity in the IITA accessions using ISSR markers was found to be the lowest, with Ne, H, and I values of 1.7166, 0.3817 and 0.5551, respectively. The genetic diversity values of these populations were ranked as EbCp > EnCp > AbCp > IITA from high to low based on polymorphisms at the selected ISSR loci.

**Table 3:** Inter-simple sequence repeat allele frequency, allele number, genetic diversity and polymorphic information content

Marker	Major allele frequency	No. of obs.	No of Allele	Gene diversity	PIC
ISSR 825	0.1667	18.0000	14.0000	0.9136	0.9073
UBC 14	0.8333	18.0000	4.0000	0.2963	0.2834
UBC 816	0.3333	18.0000	6.0000	0.7716	0.7382
UBC 826	0.3889	18.0000	8.0000	0.7778	0.7529
Mean	0.3778	18.0000	9.4000	0.7358	0.7192

*PIC* Polymorphic information content of ISSR markers used

**Table 4:** Genetic diversity parameters generated from 18 *Vigna unguiculata* using four ISSR markers

Population	Size	Ne	H	I
AbCp	5	1.7955	0.7273	0.6264
EnCp	5	1.8571	0.4580	0.6498
EbCp	5	1.9831	0.4957	0.6888
IITA	3	1.7166	0.3817	0.5551
Mean	–	1.8516	0.4498	0.6383
St. Dev	–	0.2255	0.0894	0.1026

*Ne* Effective number of alleles, *H* Nei’s gene diversity, *I* Shannon’s Information index, *AbCp Vigna unguiculata* population from Abia State, *EnCp* Population from Enugu State, *EbCp* Population from Ebonyi Sate and *IITA* Population from International Institute of Tropical Agriculture

With the four ISSR markers, the number of polymorphic loci (NPL) and percentage polymorphic loci (PPL) obtained from the 10 cowpea accessions ranged from 17 to 18 and 94.44–100, respectively (Table 5). The Ne, H and I values spanned from 1.2887–1.7831, 0.2112–0.4335 and 0.3583–0.6237 with their standard deviation ranges from 0.1940–0.1797, 0.0925–0.0600 and 0.1254–0.0639, respectively. From the ISSR markers, the mean values of Ne, H and I from the entire population were 1.8516, 0.4498 and 0.6383 with standard deviations of 0.2255, 0.0894 and 0.1026, respectively.

**Table 5:** Genetic diversity within Southern and Western Nigerian *Vigna unguiculata* using inter-simple sequence repeat markers

Marker	NPL	PPL	Ne	H	I
ISSR825	18	100.00	1.7831(0.1797)	0.4335(0.0600)	0.6237(0.0639)
UBC814	17	94.44	1.2887(0.1940)	0.2112(0.0925)	0.3583(0.1254)
UBC816	18	100.00	1.5739(0.2907)	0.3429(0.1250)	0.5184(0.1447)
UBC826	17	94.44	1.4685(0.2944)	0.2936(0.1371)	0.4552(0.1759)

Standard deviations are in parentheses, *NPL* number of polymorphic loci, *PPL* percentage polymorphic loci, mean *Ne* Effective number of alleles, mean *H* Nei’s gene diversity, mean *I* Shannon’s Information index

The genetic variation assessed using ISSR markers, revealed that the mean values of total gene diversity (Ht), gene diversity within population (Hs), coefficient of gene differentiation (Gst) and level of gene flow (Nm) were 0.4498, 0.3203, 0.2878 and 1.2371 respectively (Table 6). The Ht values ranged from 0.3817–0.4957; Hs values varied from 0.2551–0.3482; Gst from 0.2049–0.3180 and Nm from 1.2497–3.5193. The Gst value recorded 0.2878 indicating about 29% of the total genetic divergence among the populations and the remaining 71% within the populations.

**Table 6:** Genetic differentiation in the populations of *Vigna unguiculata* using ISSR markers

Population	Population size	Ht	Hs	Gst	Nm
AbCp	5	0.4364	0.3432	0.2049	3.5193
EnCp	5	0.458	0.3086	0.3180	1.2497
EbCp	5	0.4957	0.3482	0.2969	1.5252
TVu	3	0.3817	0.2551	0.2806	1.9238
Mean	–	0.4498	0.3203	0.2878	1.2371
St. Dev	–	0.008	0.0046		

*Ht* total gene diversity, *Hs* gene diversity within population, *Gst* coefficient of gene differentiation and *Nm* estimate of gene flow from Gst or Gcs. E.g., *Nm* 0.5(1 - Gst)/Gst, *AbCp* population of *Vigna unguiculata* from Abia State, *EnCp* population of *Vigna unguiculata* from Enugu State, *EbCp* population of *Vigna unguiculata* from Ebonyi State, and *TVu* population of *Vigna unguiculata* from IITA, Ibadan

### Genetic diversity revealed by start codon targeted (SCoT) polymorphic markers

**Fig. 4:**
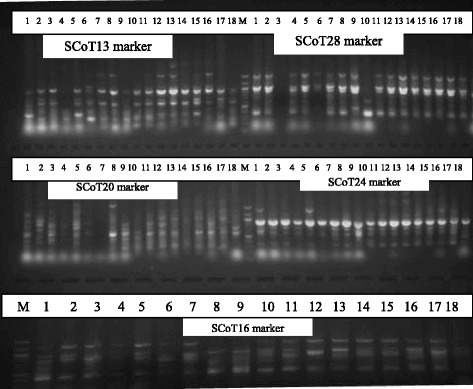
Amplification profiles of eighteen cowpea DNA samples using SCoT markers: M = 100 bp step DNA ladder, 1–5 (AbCp-Abia cowpea), 6–10 (EnCp–Enugu cowpea), 11–15 (EbCp–Ebonyi cowpea), 16–18 (IITA, Ibadan, Oyo) and L.G.A = Local government area

**Fig. 5:**
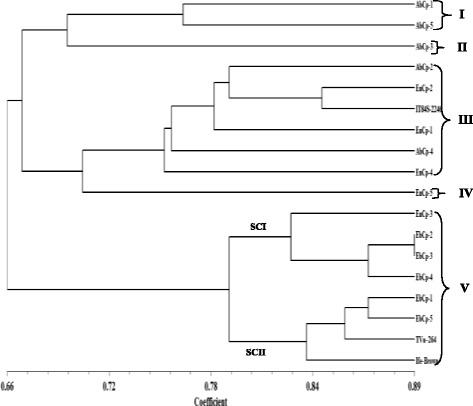
Dendrogram of 18 accessions of *Vigna unguiculata* generated with Start codon targeted markers

**Fig. 6:**
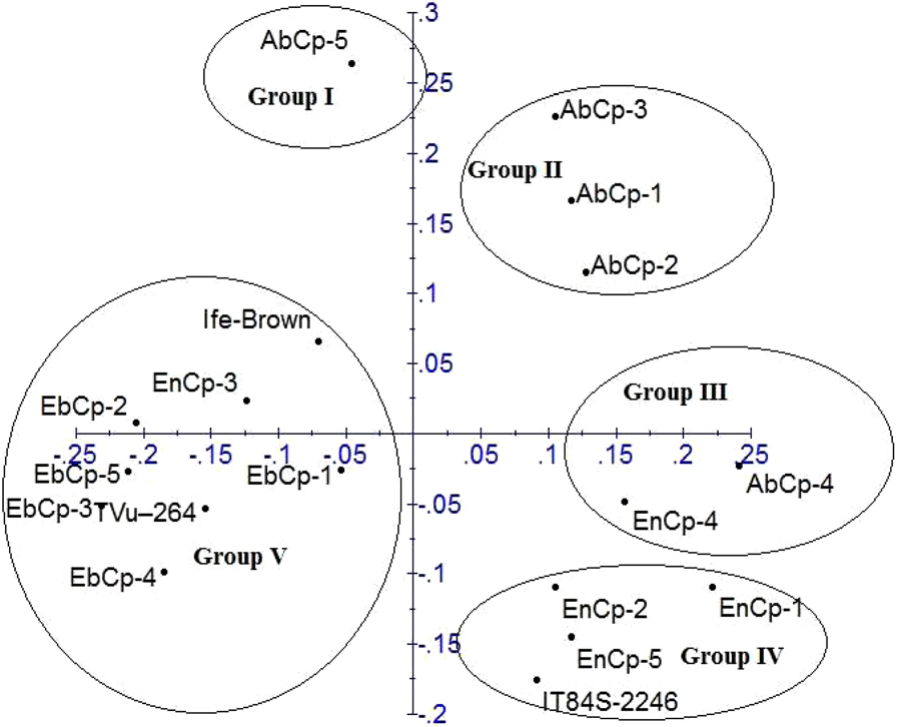
Principal component analysis of 18 *Vigna unguiculata* generated from Start codon targeted markers

Similarly, a total of 10 SCoT polymorphic markers were used to access the level of genetic diversity in the same 18 accessions of *V. unguiculata* analyzed for genetic diversity using ISSR markers to compare the powers of the two marker systems to discriminate the accessions. Out of the 10 primers used, only five (5) produced scorable bands that were used in the analysis (Fig.4). A dendrogram of the 18 accessions generated by UPGMA procedure similarly grouped the accessions into five major clusters at a similarity index of 0.72 (Fig. 5). Cluster I consisted of accessions AbCp-1 and AbCp-5 from Abia State (AbCp). Cluster II contained only accession AbCp-3 also from Abia State. Cluster III grouped together accessions AbCp-2 and AbCp-4 (Abia), EnCp-1, EnCp-2 and EnCp-4, (Enugu), and IT84S-2246 from IITA, and. Cluster IV contained only accession EnCp-5 from Enugu State while the fifth cluster was subdivided into two subclusters SCI and SCII. SCI contained accession EnCp-3 (Enugu), and accessions EbCp-2, EbCp-3 and EbCp-4 from Ebonyi with accessions EbCp-2 and EbCp-3 from Ebonyi being more closely related. Accessions EbCp-1 and EbCp-5 from Ebonyi, and TVu-264 and Ife Brown from IITA were grouped together in subcluster II (SCII). Cluster V comprised of a mixture of accessions from Enugu, Ebonyi and IITA. Principal component analysis of the generated amplicons from SCoT markers resulted in five clusters (Fig. 6). Each cluster is a representative of unique accession or accessions.

The five (5) SCoT markers amplified a total of 52 alleles. The number of alleles amplified by each marker ranged from 8 to 15, with a mean of 11.1667 (Table 7). Polymorphic information content (PIC) values ranged from 0.6364–0.9210 with a mean value of 0.8009. The SCoT markers SCoT13, SCoT28, SCoT20 and SCoT24 were polymorphic while SCoT16 was monomorphic with the least PIC value of 0.6304. The genetic diversity ranged values from 0.6543–0.9259 with a mean value of 0.8158, while major allele frequency spanned from 0.1111–0.5556 with a mean value of 0.3148. Allelic scores, counts and frequencies obtained from the accessions of *V. unguiculata* using SCoT markers were shown (Additional file 2: Table S2). These SCoT markers also revealed the highest genetic diversity in Ebonyi cowpea (EbCp) population compared to the other populations, with Ne, H, and I values of 1.9000, 0.4958 and 0.6889, respectively (Table 8), while the genetic diversity in Enugu (EnCp) population was found to be the lowest, with corresponding values of 1.8853, 0.4686 and 0.6613. The genetic diversity values of these populations were ranked in a decreasing order as EbCp > IITA > AbCp > EnCp. From the five SCoT markers data, the number of polymorphic loci (NPL) and percentage polymorphic loci (PPL) ranged from 17 to 18 and 94.44–100, respectively (Table 9). The Ne, H and I values spanned from 1.7416–1.9181, 0.4111–0.4778 and 0.5911–0.6706 with their standard deviation ranges from 0.2422–0.0776, 0.1152–0.0226 and 0.1578–0.0233, respectively. From the SCoT polymorphic markers, the mean values of Ne, H and I from the entire population were 1.9298, 0.4808 and 0.6736 with standard deviations of 0.0876, 0.0248 and 0.0254, respectively. The genetic variation assessed using ISSR markers, revealed that the mean values of total gene diversity (Ht), gene diversity within population (Hs), coefficient of gene differentiation (Gst) and level of gene flow (Nm) were 0.4808, 0.4522, 0.0594 and 7.9245 respectively (Table 10). The Ht values ranged from 0.4686–0.4958; Hs values varied from 0.4344–0.4680; Gst from 0.0543–0.0735 and Nm from 9.3067–419.6100. The Gst value recorded 0.0594 indicating about 6% of the total genetic divergence among the populations and the remaining 94% within the populations.

**Table 7:** Start codon targeted allele frequency, allele number, genetic diversity and polymorphic information content

Marker	Major allele frequency	No. of observation	No of Allele	Gene diversity	PIC
SCoT13	0.1111	18.0000	15.0000	0.9259	0.9210
SCoT28	0.5000	18.0000	8.0000	0.7037	0.6792
SCoT20	0.1667	18.0000	14.0000	0.9136	0.9073
SCoT24	0.3889	18.0000	8.0000	0.7778	0.7529
SCoT16	0.5556	18.0000	7.0000	0.6543	0.6304
Mean	0.3148	18.0000	11.1667	0.8158	0.8009

*PIC* Polymorphic information content

**Table 8:** Genetic diversity parameters generated from 18 accessions of *Vigna unguiculata* using SCoT markers

Population	Size	Ne	H	I
AbCp	5	1.9100	0.4747	0.6673
EnCp	5	1.8853	0.4686	0.6613
EbCp	5	1.9837	0.4958	0.6889
IITA	3	1.9473	0.4859	0.6788
Mean	–	1.9298	0.4808	0.6736
St. Dev	–	0.0876	0.0248	0.0254

*Ne* Effective number of alleles, *H* Nei’s gene diversity, *I* Shannon’s Information index *AbCp Vigna unguiculata* population from Abia State, *EnCp* Population from Enugu State, *EbCp* Population from Ebonyi Sate and *IITA* Population from International Institute of Tropical Agriculture

**Table 9:** Genetic diversity within Southern Nigerian and Western *Vigna unguiculata* using start codon targeted markers

Marker	NPL	PPL	Ne	H	I
SCoT13	18	100.00	1.8776(0.1337)	0.4644(0.0442)	0.6564(0.0471)
SCoT28	17	94.44	1.8110(0.2426)	0.4333(0.1168)	0.6145(0.1604)
SCoT20	17	94.44	1.7416(0.2422)	0.4111(0.1152)	0.5911(0.1578)
SCoT24	18	100.00	1.9070(0.0900)	0.4744(0.0264)	0.6671(0.0272)
SCoT16	18	100.00	1.9181(0.0776)	0.4778(0.0226)	0.6706(0.0233)

Standard deviations are in parentheses, *NPL* number of polymorphic loci, *PPL* percentage polymorphic loci, mean *Ne* Effective number of alleles, mean *H* Nei’s gene diversity, mean *I* Shannon’s Information index

**Table 10:** Genetic differentiation in the populations of *Vigna unguiculata* using SCoT markers

Population	Population size	Ht	Hs	Gst	Nm
AbCp	5	0.4747	0.4488	0.0543	419.6100
EnCp	5	0.4686	0.4344	0.0735	20.2960
EbCp	5	0.4958	0.4680	0.0561	9.3067
TVu	3	0.4859	0.4613	0.0495	20.3542
Mean	–	0.4808	0.4522	0.0594	7.9245
St. Dev	–	0.0006	0.001		

*Ht* total gene diversity, *Hs* gene diversity within population, *Gst* coefficient of gene differentiation and *Nm* estimate of gene flow from Gst or Gcs. E.g., *Nm* 0.5(1 - Gst)/Gst, *AbCp* population of *Vigna unguiculata* from Abia State, *EnCp* population of *Vigna unguiculata* from Enugu State, *EbCp* population of *Vigna unguiculata* from Ebonyi State, and *TVu* population of *Vigna unguiculata* from IITA, Ibadan

### Comparison of ISSR and SCoT data obtained from the 18 *Vigna unguiculata* accessions

Five major groups and five clusters each were obtained from the dendrograms and PCAs of ISSR and SCoT data (Table 11). Mean major allele frequency of 0.3778 was detected with the ISSR while that of the SCoT markers was 0.3148. Total number of alleles was 32 with a mean of 9.4 for the ISSR while that of SCoT was 52 with an average value of 11.1667. The ISSR marker had a mean genetic diversity value of 0.7358, while SCoT had 0.8158. The mean PIC in ISSR was identified to be 0.7192, while that of the SCoT was 0.8009. The allele range count was 1–15 in ISSR marker, while SCoT had a range of 1–10. Allele frequency ranges were 0.0556–0.8333 and 0.0556–5556 in ISSR and SCoT, respectively. The total number of polymorphic loci of 70 and 80 were obtained from ISSR and SCoT, respectively. Range of percentage polymorphic loci of 94.44–100 was detected in both markers. Ranges of Ne were 1.2887 ± 0.1797–1.7831 ± 0.2944 and 1.7416 ± 0.0776–1.9181 ± 0.2426 in ISSR and SCoT, respectively. The values of H ranged from 0.2112 ± 0.0600–0.4335 ± 0.1371 and 0.4111 ± 0.0226–0.4778 ± 0.1168 in ISSR and SCoT, respectively. The values of I obtained from ISSR and SCoT markers were 0.3583 ± 0.0639–0.6237 ± 0.1759 and 0.5911 ± 0.0233–0.6706 ± 0.1604. Also, Ht recorded in the ISSR marker data ranged from 0.3817–0.4957 and 0.4686–0.4958 in SCoT. The values of Hs in ISSR spanned from 0.2551–0.3482 and SCoT had 0.4344–0.4680. In ISSR marker data, the Gst ranged from 0.2049–0.3180 and 0.0543–0.0735 in SCoT, while the Nm obtained with the ISSR marker data ranged from 1.2497–3.5193 and 9.3067–419.6100 for the SCoT markers.

**Table 11:** Comparative analysis between inter-simple sequence repeat and start codon targeted polymorphic markers

Parameters	Markers	
	ISSR	SCoT
No of clusters from dendrogam	5	5
No of clusters from PCA	5	5
Mean major allele frequency	0.3778	0.3148
No. of observations	18	18
Total no of alleles	32	52
Mean no of alleles	9.4000	11.1667
Mean gene diversity	0.7358	0.8158
Mean PIC	0.7192	0.8009
Allele count range	1–15	1–10
Allele frequency range	0.0556–0.8333	0.0556–0.5556
Total No of polymorphic loci	70	80
Range of % polymorphic loci	94.44–100.00	94.44–100.00
Range of effective alleles, Ne	1.2887–1.7831(0.1797–0.2944)	1.7416–1.9181(0.0776–0.2426)
Nei’s genetic diversity, H	0.2112–0.4335(0.0600–0.1371)	0.4111–0.4778(0.0226–0.1168)
Shannon’s information index, I	0.3583–0.6237(0.0639–0.1759)	0.5911–0.6706(0.0233–0.1604)
Total gene diversity, Ht	0.3817–0.4957	0.4686–0.4958
Gene diversity in population Hs	0.2551–0.3482	0.4344–0.46800
Coeff of gene differentiation Gst	0.2049–0.3180	0543–0.0735
Level of gene flow, Nm	1.2497–3.5193	9.3067–419.6100

*PIC* Polymorphic information content, Standard deviations are in parentheses

## Discussion

Sustainability in food security within sub-Saharan Africa and Nigeria in particular, is dependent on development, improvement and preservation of crops, especially at this critical global threat to life occasioned by worsening climatic conditions [36]. It has been established that cowpea possesses the characteristic high variability, drought tolerance and adaptability to support food security [37–39]. Therefore, genetic diversity study using informative markers is critical in the management, genetic improvement, identification of unique genotypes/accessions and utilization of germplasm [40–42]. In this study, ISSR and SCoT markers were used to assess the degree of genetic diversity and relationship among the accessions of *V. unguiculata* within the Southern and Western Nigeria. Comparing different markers for genetic diversity studies can provide a more informative classification than a single method alone [43]. Our results show both markers have strong discriminating power on the cowpea accessions as indicated by the high values of the genetic diversity indices including number of alleles per primer, PIC values, percent polymorphism, among others. The markers differentiated the accessions into five major groups or clusters which is in agreement with the previous work in which SCoT and ISSR markers were used to study the genetic diversity of 38 grain legume crop, annual *Cicer* species, though SCoT identified four groups contrary to five groups detected in this study [44]. Also, five clusters have been reported in 35 *Vigna* accessions using ISSR markers [45].

Some of the accessions got clustered with IITA materials (TVu-264, Ife Brown and IT84S-2246) popularly used as breeding and research materials due to possession of unique traits. The mean activities on seed beetle, *Callosobruchus maculatus* (F.) using Ife Brown and IT84S-2246 varieties were reported show susceptibility and resistance, respectively [46, 47]. This suggests that *V. unguiculata* accessions identified in the same cluster were more genetically similar while those found on different clusters might be more genetically diverse. The farther away accessions are from each other, the more the possibility of possessing wider genetic diversity which also reflects their locations on clusters as previously reported [48]. It has been noted that populations having high genetic diversity of neutral markers and alleles could be utilized as suitable candidates for high adaptive variation, fitness and conservation [49–51]. From previous studies, allelic richness is an indicator of genetic diversity and it is mostly used in assessment of molecular markers to identify populations for conservation and breeding purpose [51, 52]. In this study, both marker types demonstrated high values for total number of alleles mean numbers of alleles, PIC, genetic diversity and total number of polymorphic loci with higher values in SCoT markers in comparison with ISSR. This showed that SCoT markers may be more efficient in studying genetic diversity and relatedness of *V. unguiculata*. The total number of alleles (ISSR: 4–14; SCoT: 7–15) and PIC (ISSR: 0.2834–0.9073; SCoT: 0.6304–0.9210) values obtained were in agreement with the values reported by Amirmoradi et al. [44] in 38 accessions of grain legume crop (annual *Cicer* species). A research on a legume crop, chickpea, using 9 SCoT markers revealed as high as 145 alleles and PIC range of 0.43–0.47 in 48 genotypes [53] and this contracts the number of alleles obtained in this study possibly due to the larger number of genotypes and species used. According to Singh et al. [45], 83 alleles were identified in 35 *Vigna* species using 17 ISSR markers but in this study, as high as 70 alleles were detected with just 4 ISSR markers. The variations in the number of alleles and PIC range observed in this study (SCoT: 80 alleles; 0.6304–0.9210 PIC) with respect to earlier reports in the literature could be attributed to different crop species and number of genotypes involved. However, it is interesting that both the ISSR and SCoT markers used in this work identified as high as 94–100 percentage polymorphic loci (% polymorphism) in the accessions studied. These values are very close to the earlier reports by Amirmoradi et al. [44] wsho reported 85.7–100% and 86.6–100% in ISSR and SCoT, respectively. Zhang et al. [54] reported similar values (ISSR: 82.35–96%; SCoT: 81.25–100%) in a different plant species called Switch grass. Many other reports with other marker systems showed lower percent polymorphisms relative to our findings in this study. For instance, RAPD and microsatellite markers revealed 71.2% [52], 90.0% [55], 55.5% [56], 46.5% [57, 58], 64.5% [59] and 58.44% polymorphism [30]. The percentage polymorphism obtained from this present study is quite high indicating the higher informative nature of the markers used (ISSR and SCoT). It has been revealed that high polymorphism identifiable by molecular markers was hinged on the presence of repeated sequences of AC, CA, AG and GA [30]. It has been observed that genetic parameters including Nei’s genetic diversity, numbers of effective alleles as well as Shannonʼs information index are very crucial in the study of genetic diversity in plant species [60, 61]. They are essential measures of degree of genetic diversity. Among the populations of *V. unguiculata* accessions evaluated in this study, we found that the Nei’s genetic diversity (Ne), effective number of alleles (H) and Shannonʼs information index (I) were highest in Ebonyi population followed by Enugu and Abia (all in the Southeastern Nigeria), while the least diverse was the IITA population. In assessing the populational diversity of the accessions of *V. unguiculata*, the ones from Ebonyi state demonstrated the highest values of Ht and Hs in both ISSR (Ht = 0.4957; Hs = 0.3482) and SCoT (Ht = 0.4958; Hs = 0.4680) markers. Generally in ISSR data matrix, all the assessed parameters including Ht, Hs, Gst and Nm were higher in SCoT compared to the ones obtained with ISSR markers, except the Gst that was identified to be greater in all the populations analysed with ISSR markers.

## Conclusion

This finding suggests that accessions from Ebonyi State are probably more genetically distinct and may not have been subjected to hybridization with other accessions and might have originated from various ancestors of cowpea in the past as opined by other researchers [52, 62]. The study also revealed that Start codon targeted (SCoT) markers are more efficient in resolving genetic diversity and relatedness than ISSR in cowpea. This is indicated by the higher values of Ne, H, I, Ht, Hs and Nm obtained with SCoT markers compared to those obtained with ISSR markers. The SCoT markers generally exhibited higher values of all the diversity parameters evaluated than ISSR markers, except in major allele frequency, allele count, allele frequency and gene flow. This finding demonstrates that these exceptional parameters may not be good indicators of genetic diversity in *V. unguiculata* species. The comparative genetic diversity in the accessions of *V. unguiculata* from Southern Nigeria using ISSR and SCoT polymorphic markers demonstrated the high efficiencies of both markers in discriminating cowpea accessions with SCoT markers being more efficient than ISSR markers. Cowpea accessions from Ebonyi were found to be most genetically diverse compared to those obtained from other States and IITA. In this study, ISSR and SCoT markers have proved to be efficient for genetic fingerprinting and other potential applications including determination of seed purity, efficient utilization and management of genetic resources in cowpea. They played major roles in the identification of diverse accessions collected by linking some to already known IITA breeding and research accessions (Ife Brown, TVu-264 and IT84S-2246) in which some have been known to be susceptible or resistant to some disease attacks. These can be utilized when making a choice for hybridization to provide genetic barriers against different biotic stressors to improve cowpea breeding. Thus, the results obtained in this study demonstrated a wide genetic diversity of the cowpea accessions. Based on the outcome of this study, we suggest that selection could be made from the accessions in each of the unique clusters detected by the polymorphic SCoT markers as stock for novel gene exchange in crop breeding.

## Additional files

Additional file 1: Table S1. Allelic scores, count and frequencies obtained from *Vigna unguiculata* accessions using Inter-simple sequence repeat (ISSR) markers. (DOC 58 kb)

Additional file 2: Table S2. Allelic scores, count and frequencies obtained from *Vigna unguiculata* accessions using Start codon targeted (SCoT) markers. (DOC 73 kb)

## Abbreviations

AFLP: Amplified fragment length polymorphism
BRDC: Biotechnology Research and Development Center
DEPC: Diethyl pyrocarbonate
Gs: Coefficient of differentiation
H: Nei’s genetic diversity
Hs: Gene diversity within population
Ht: Total genetic diversity among population
I: Shannon’s information index
IITA: International Institute of Tropical Agriculture
ISSR: Inter-simple sequence repeat
Ne: Effective number of alleles
Nm: Level of gene flow
NPL: Number of polymorphic loci
PCR: Polymerase chain reaction
PIC: Polymorphic information content
PPL: Percentage polymorphic loci
RAPD: Random amplified polymorphic DNA
SCI: Subcluster I
SCII: Subcluster II
SCoT: Start codon targeted
SSR: Simple sequence repeat
UPGMA: Unweighted pair group mean with arithmetic

## References

[1] Singh SR (1999). Van emdem HF. Insect pests of grain legumes. Annu Rev Entomol.

[2] Saidi M, Ngouajio M, Itulya FM, Ehlers J (2007). Leaf harvesting initiation time and frequency affect biomass partitioning and yield of cowpea. Crop Sci.

[3] Agbogidi OM, Egho EO (2012). Evaluation of eight varieties of cowpea (*Vigna unguiculata* (L.) Walp) in Asabaagro-ecological environment, Delta state, Nigeria. Eur J Sustain Dev.

[4] Quin FM. Importance of cowpea. In: Advances in cowpea research. B.B. Singh, K.E. Dashiell, D.R Mohan Raj and L.E.N. Jackai (Eds.), pp. 10-12. Printed by Colcorcraft, Hong Kong, 1997; 375pp.

[5] Bashir M, Ahamad Z, Ghafoor A (2002). Cowpea aphid-borne mosaic potyvirus: a review. Int J Pest Mgt.

[6] Boukar O, Massawe F, Muranaka S, Franco J, Maziya-Dixon B, Singh B, Fatokun C (2011). Evaluation of cowpea germplasm lines for protein and mineral concentrations in grains. Pl Gen Res..

[7] FAO/INFOODS (2012) Guidelines for checking food composition data prior to the publication of a user table/Database-version 1.0. Food and Agricultural Organisation/International Network of Food Data System, Rome. Available at http://www.fao.org/fileadmin/templates/foodcomposition/documents/upload/Guidelines_data_checking.pdf (Accessed Nov., 2012).

[8] Clark AJ, Meisinger JJ, Decker AM, Mulford FR (2007). Effects of a grass-selective herbicide in a vetch-rye cover crop system on nitrogen management. J Agron.

[9] Boukar O, Fatokun CA, Roberts PA, Abberton M, Huynh BL, Close TJ, Boahen SK, Higgins TJV, Ehlers JD, De Ron AM (2015). Cowpea, in grain legumes, series handbook of plant breeding.

[10] Tarawali SA, Singh BB, Peters M, Blade SP. Cowpea haulms as fodder. In: Singh BB, Mohan Raj DR, Dashiel KE, Jackai LEN, editors. In advances in cowpea research. Copublication of International Institute of Tropical Agriculture (IITA) and Japan International Research Center for Agricultural Sciences (JIRCAS). Ibadan: IITA; 1997. pp. 313-25.

[11] Abayomi YA, Ajibade TV, Salawudeen BF, Samuel OF (2008). Growth and yield resopnse of cowpea (*Vigna unguiculata* (L.) Walp) genotypes to nitrogen fertilizer (NPK) application in the southern guinea savanna zone of Nigeria. Asian J Pl Sci.

[12] Asiwe JAN, Belane A, Dakora FD. Evaluation of cowpea breeding lines for nitrogen fixation at ARC- grain crops institute, Potchefstroom, South Africa. Paper presented at the 16^th^ international congress on biological nitrogen fixation. Montana, USA; 2009. p. pp14–9.

[13] FAO. World Agriculture: 2002; towards 2015/2030*.* Summary report, FAO, Rome.

[14] Adaji MJ, Olufala OO, Aliyu L. Effect of intra-row spacing and stand density on the growth and yield of cowpea (*Vigna unguculata* (L.) Walp). In: Olulaja, O. O., Omokore, D.F., Akpa, G. N. and Sanni, S. A. (eds.). Proceedings of the 41st annual conference of the agricultural Society of Nigeria (ASN) held at the Institute for Agricultural Research, Samaru, Ahmadu Bello University, Zaria between 22nd and 26th October. 2007; 153– 157pp.

[15] FAO (2008). *Agricultural Production Status*. Food and agricultural organisation.

[16] TJAI (2010). Cowpea: a versatile legume for hot, dry conditions. Columbia, MO. Thomas Jefferson agricultural institute.

[17] FAOSTAT. FAO Statistics on-line website, http://faostat3.fao.org/home/E (accessed: 27 October 2015).

[18] Egho EO. Control of major insect pests of cowpea *(Vigna unguiculata (L.)* Walp using conventional and non-conventional chemicals. A Ph.D Thesis submitted to the Department of Agronomy, Delta State University, Asaba Campus. 2009; pp224.

[19] Ehlers JD, Hall AE (1997). Cowpea (*Vigna unguiculata* L. Walp). Field Crops Res.

[20] Kareem KT, Taiwo MA (2007). Interactions of viruses in cowpea: effects on growth and yield parameters. Viro J.

[21] Souleymane A, Aken’Ova AME, Fatokun CA, Alabi OY (2013). Screening for resistance to cowpea aphid (*Aphis craccivora* Koch) in wild and cultivated cowpea (*Vigna unguiculata* L. Walp) accessions. Int J Sci Env Tech.

[22] Singh BB, Ehlers JD, Sharma B, Freire FFR, Fatokun CA, Tarawali SA, Singh BB, Kormawa PM, Tamo M (2002). Recent progress in cowpea breeding. Challenges and opportunities for enhancing sustainable cowpea production.

[23] Muchero W, Diop NN, Bhat PR. A consensus genetic map of cowpea [*Vigna unguiculata* (L) Walp.] and synteny based on EST-derived SNPs. Proceeding of National Academy of Science, USA 2009; 106(43):18159– 18164. 10.1073/pnas.0905886106 PMC2761239 19826088

[24] Akibonde S, Maredia M (2011). East Lansing, MI, USA: Department of Agricultural, food and resource economics, Michigan State University. Global and regional trends in production, trade and consumption of food legume crops.

[25] Varshney RK, Bansal KC, Aggarwal PK, Datta SK, Craufurd PK (2011). Agricultural biotechnology for crop improvement in a variable climate: hope or hype?. Trends in Pl Sci.

[26] Lake IR, Hooper L, Abdelhamid A, Bentham G, Boxall AB, Draper A, Fairweather-Tait S, Hulme M, Hunter PR, Nichols G, Waldron KW (2012). Climate change and food security: health impacts in developed countries. Env Health Perspec.

[27] Fang J, Devanand PS, Chao CCT. Poster section 4-genetics and germplasm, 102nd annual conference of International conference of the America Society of Horticultural Sciences. In: Genetics diversity of cowpea [*Vigna unguiculata* (L.) Walp] breeding lines from different countries determined by AFLP markers. Las Vegas; 2005.

[28] Varshney RK, Chabane K, Hendre PS, Aggarwal RK, Graner A. Comparative assessment of EST-SSR, EST-SNP and AFLP markers for evaluation of genetic diversity and conservation of genetic resources using wild, cultivated and elite barleys. Pl Sci 2007; 173: 638–649.

[29] Asare AT, Gowda BS, Galyuon IKA, Aboagye LM, Takrama JF, Timko MP (2010). Assessment of the genetic diversity in cowpea (*Vigna unguiculata* (L.) Walp) germplasm from Ghana using simple sequence repeat markers. Pl Gen Res.

[30] Ghalmi N, Malice M, Jacquemin JM, Ounane SM, Mekliche L, Baudoin JP (2010). Morphological and molecular diversity within Algerian cowpea (*Vigna unguiculata* (L.) Walp.) landraces. Gen Res Crop Evo.

[31] Malviya N, Yadav D, Sarangi BK, Yadav MK (2012) Analysis of genetic diversity in cowpea (Vigna Unguiculata L. Walp.) cultivars with random amplified polymorphic DNA markers. Pl System Evo. 2012; 29: 523-526.

[32] Hegde VS, Mishra SK (2009). Landraces of cowpea, *Vigna unguiculata* (L.) Walp. As potential sources of genes for unique characters in breeding. Genet Res Crop Evol.

[33] Collard BCY, Mackill DJ (2009). Start codon targeted (SCoT) polymorphism: a simple, novel DNA marker technique for generating gene targeted markers in plants. Pl Mol Bio Report.

[34] Zietkiewicz E, Rafalski A, Labuda D. Genome fingerprinting by simple sequence repeats (SSR)-anchored polymerase chain reaction amplification. Genom 1994; 20: 176-183. 10.1006/geno.1994.1151 8020964

[35] Ojuederie OB, Balogun MO, Fawole I, Igwe DO, Olowolafe MO (2014). Assessment of the genetic diversity of African yam bean (*Sphenostylis stenocarpa* Hochst ex. A. Rich harms) accessions using amplified fragment length polymorphism (AFLP) markers. Afr J Biotech.

[36] Udensi O, Ikpeme EV, Edu EA, Ekpe DE (2012). Relationship studies in cowpea (*Vigna unguiculata* L. Walp) landraces grown under humid lowland condition. Int. J Agric Res.

[37] Tharanathan RN, Mahadevamma S (2003). Grain legumes: a boon to human nutrition. Trends Food Sci Technol.

[38] Costa GEDA, Queiroz-Monici KDS, Reis SMPM, de Oliveira AC (2006). Chemical composition, dietary fibre and resistant starch contents of raw and cooked pea, common bean, chickpea and lentil legumes. Food Chem.

[39] Udensi O, Edu EA, Umana UJ, Ikpeme EV (2011). Estimation of genetic variability in locally grown pulses (*Cajans cajan* (L.) Millsp and *Vigna unguiculata* (L.) Walp): a panacea for sourcing superior genotypes. Pak J Biol Sci.

[40] Peleg Z, Saranga Y, Krugman T, Abbo S, Nevo E, Fahima T (2008). Allelic diversity associated with aridity gradient in wild emmer wheat populations. Plant Cell Environ.

[41] Nevo E, Fu YB, Pavlicek T, Khalifa S, Tavasi M, Beiles A (2012). Evolution of wild cereals during 28 years of global warming in Israel. Proc Natl Acad Sci U S A.

[42] Rashid J, Tamanna FM, Hossain MAR, Alam MS (2012). Genetic variation in endangered butter catfish, *Ompok bimaculatus* (bloch) populations revealed by random amplified polymorphic DNA (RAPD) fingerprinting. Int J Biosci.

[43] Souframanien J, Gopalakrishna T (2004). A comparative analysis of genetic diversity in blackgram genotypes using RAPD and ISSR markers. Theory Appl Gen.

[44] Amirmoradi B, Talebi R, Karami E (2012). Comparison of genetic diversity and differentiation among annual Cicer species using start codon targeted (SCoT) polymorphism, DAMD-PCR, and ISSR markers. Pl Sys Evo Springer.

[45] Singh A, Dikshit HK, Jain N, Singh D, Yadav RN (2014). Efficiency of SSR, ISSR and RAPD markers in molecular characterization of mungbean and other *Vigna* species. India J Biotech.

[46] Ofuya TI, Credland PF (1995). Responses of three populations of the seed beetle, *Callosobruchus maculatus* (F.) (Coleoptera: Bruchidae), to seed resistance in selected varieties of cowpea, *Vigna unguiculata* (L.) Walp. J Stored Prod Res.

[47] Fatokun CA, Tarawali SA, Singh BB, Kormawa PM, Tamò M (2002). Challenges and opportunities for enhancing sustainable cowpea production. Proceedings of the world cowpea conference III held at the International Institute of Tropical Agriculture (IITA), Ibadan, Nigeria, 4–8 September 2000.

[48] Skroch PW, Nienhuis J (1995). Qualitative and quantitative characterization of RAPD variation among snap bean (*Phaseolus vulgaris*) genotypes. Theor Applied Genet.

[49] Van Zonneveld M, Scheldeman X, Escribano P, Viruel MA, van Damme P, Garcia W (2012). Mapping genetic diversity of cherimoya (*Annona cherimola* mill.) and application of spatial analysis of conservation and use of plant genetic. PLoS One.

[50] Ilves A, Lanno K, Sammul M, Tali K (2013). Genetic variability, population size and reproduction potential in *Ligularia sibirica* (L.) populations in Estonia. Conserv Gen.

[51] Vinceti B, Loo J, Gaisberger H, Van Zonneveld MJ, Schueler S, Konrad H (2013). Conservation priorities for *Prunus africana* defined with the aid of spatial analysis of genetic data and climatic variables. PLoS One.

[52] Patil DM, Sawardekar SV, Gokhale NB, Bhave SG, Sawant SS (2013). Genetic diversity analysis in cowpea [*Vigna unguiculata* (L.) Walp.] by using RAPD markers. Int J Innov Biotechnol Biochem.

[53] Hajibarat Z, Saidi A, Hajibarat Z, Talebi R (2015). Characterization of genetic diversity in chickpea using SSRmarkers, start Codon targeted polymorphism (SCoT) and conserved DNA-derived polymorphism (CDDP). Phy Mol Bio Pl.

[54] Zhang Y, Yan H, Jiang X, Wang X, Huang L, Xu B, Zhang X, Zhang L (2016). Genetic variation, population structure and linkage disequilibrium in Switchgrass with ISSR. SCoT and EST-SSR markers BioMed Central.

[55] Prasanthi L, Geetha B, Jyothi BNR, Reddy KR (2012). Evaluation of genetic diversity in cowpea, *Vigna unguiculata* (L.) Walp gentotypes using random amplified polymorphic DNA (RAPD). Curr Biotica.

[56] Khan IA, Kamineni V, Poornima S, Jahan P, Hasan Q, Rao P (2015). Tumor necrosis factor alpha promoter polymorphism studies in pregnant women. J Reprod Health Med.

[57] Pandey A, Roca MG, Read ND, Glass NL (2004). Role of a mitogen-activated protein kinase pathway during conidial germination and hyphal fusion in *Neurospora crassa*. Eukaryot Cell.

[58] Zannou A, Kossou DK, Ahanchede A, Zoundjihekpon J, Agbicodo E, Struik PC, Sanni A (2008). Genetic variability of cultivated cowpea in Benin assessed by random amplified polymorphic DNA. Afr J Biotechnol.

[59] Sharawy WM, El-Fiky ZA (2003). Characterization of cowpea (*Vigna unguiculata* L.) genotypes based on yield traits and RAPD-PCR analysis. Arab J Biotechnol.

[60] Hamilton M (2009). Population genetics.

[61] Freeland J, Kirk H, Petersen S (2011). Molecular Ecology.

[62] Ba FS, Pasquet RS, Gepts P (2004). Genetic diversity in cowpea [*Vigna unguiculata* (L.) Walp.] as revealed by RAPD markers. Genet Resour Crop Evol.

