# Supplementary material for: Assessment of genetic diversity in Vigna unguiculata L. (Walp) accessions using inter-simple sequence repeat (ISSR) and start codon targeted (SCoT) polymorphic markers
Source: BMC Genet. 2017 Nov 17;18:98. doi: 10.1186/s12863-017-0567-6 (PMC5693802; doi:10.1186/s12863-017-0567-6)
Supplement: Supplementary file 1 — Allelic scores, count and frequencies obtained from Vigna unguiculata accessions using Inter-simple sequence repeat (ISSR) markers. (DOC 58 kb) [file 12863_2017_567_MOESM1_ESM.doc]

**Table S1: Allelic scores, count and frequencies obtained from *Vigna unguiculata* accessions using Inter-simple sequence repeat (ISSR) markers**

| **Marker** | **Allele** | **Count** | **Frequency** |
| --- | --- | --- | --- |
| **ISSR 825** | 0/0/0/0/0/0/1/1 | 1 | 0.0556 |
| **ISSR 825** | 0/0/0/0/0/1/1/1 | 1 | 0.0556 |
| **ISSR 825** | 0/0/0/0/1/0/0/1 | 2 | 0.1111 |
| **ISSR 825** | 0/0/0/0/1/1/0/1 | 1 | 0.0556 |
| **ISSR 825** | 0/0/0/1/0/1/1/1 | 1 | 0.0556 |
| **ISSR 825** | 0/0/0/1/1/0/1/1 | 1 | 0.0556 |
| **ISSR 825** | 0/0/1/0/0/1/1/1 | 1 | 0.0556 |
| **ISSR 825** | 0/0/1/1/0/1/0/1 | 1 | 0.0556 |
| **ISSR 825** | 0/0/1/1/1/1/0/1 | 1 | 0.0556 |
| **ISSR 825** | 1/0/1/1/0/1/0/1 | 1 | 0.0556 |
| **ISSR 825** | 1/0/1/1/0/1/1/1 | 1 | 0.0556 |
| **ISSR 825** | 1/0/1/1/1/1/0/1 | 3 | 0.1667 |
| **ISSR 825** | 1/1/1/1/1/0/0/1 | 1 | 0.0556 |
| **ISSR 825** | 1/1/1/1/1/1/0/1 | 2 | 0.1111 |
| **UBC 14** | 0/0/0/0/0 | 1 | 0.0556 |
| **UBC 14** | 0/1/0/0/0 | 15 | 0.8333 |
| **UBC 14** | 0/1/1/0/0 | 1 | 0.0556 |
| **UBC 14** | 1/1/0/1/1 | 1 | 0.0556 |
| **UBC 816** | 0/0/1/0 | 1 | 0.0556 |
| **UBC 816** | 1/0/0/0 | 6 | 0.3333 |
| **UBC 816** | 1/0/0/1 | 2 | 0.1111 |
| **UBC 816** | 1/0/1/1 | 5 | 0.2778 |
| **UBC 816** | 1/1/0/1 | 2 | 0.1111 |
| **UBC 816** | 1/1/1/1 | 2 | 0.1111 |
| **UBC 826** | 0/0/0/0/0/0/0/0/0 | 1 | 0.0556 |
| **UBC 826** | 0/0/0/0/0/0/1/1/0 | 1 | 0.0556 |
| **UBC 826** | 0/1/0/1/0/1/1/1/0 | 1 | 0.0556 |
| **UBC 826** | 0/1/1/1/0/0/1/1/0 | 1 | 0.0556 |
| **UBC 826** | 1/1/1/1/0/0/1/1/0 | 3 | 0.1667 |
| **UBC 826** | 1/1/1/1/0/1/1/1/0 | 3 | 0.1667 |
| **UBC 826** | 1/1/1/1/1/0/1/1/1 | 1 | 0.0556 |
| **UBC 826** | 1/1/1/1/1/1/1/1/0 | 7 | 0.3889 |
