# Supplementary material for: Assessment of genetic diversity in Vigna unguiculata L. (Walp) accessions using inter-simple sequence repeat (ISSR) and start codon targeted (SCoT) polymorphic markers
Source: BMC Genet. 2017 Nov 17;18:98. doi: 10.1186/s12863-017-0567-6 (PMC5693802; doi:10.1186/s12863-017-0567-6)
Supplement: Supplementary file 2 — Allelic scores, count and frequencies obtained from Vigna unguiculata accessions using Start codon targeted (SCoT) markers. (DOC 73 kb) [file 12863_2017_567_MOESM2_ESM.doc]

**Table S2: Allelic scores, count and frequencies obtained from *Vigna unguiculata* accessions using Start codon targeted (SCoT) markers**

| **Marker** | **Allele** | **Count** | **Frequency** |
| --- | --- | --- | --- |
| **SCoT13** | 0/0/0/0/0/0/1/0/1/1 | 1 | 0.0556 |
| **SCoT13** | 0/0/0/1/0/0/0/1/0/1 | 1 | 0.0556 |
| **SCoT13** | 0/0/0/1/0/0/1/0/1/1 | 1 | 0.0556 |
| **SCoT13** | 0/0/0/1/0/1/0/1/1/0 | 1 | 0.0556 |
| **SCoT13** | 0/0/0/1/0/1/1/0/1/0 | 2 | 0.1111 |
| **SCoT13** | 0/0/0/1/1/1/0/1/0/1 | 1 | 0.0556 |
| **SCoT13** | 0/0/0/1/1/1/1/0/0/1 | 1 | 0.0556 |
| **SCoT13** | 0/0/0/1/1/1/1/0/1/1 | 2 | 0.1111 |
| **SCoT13** | 0/0/1/1/1/1/1/0/1/0 | 1 | 0.0556 |
| **SCoT13** | 0/1/0/1/0/1/1/0/1/1 | 2 | 0.1111 |
| **SCoT13** | 0/1/0/1/1/1/1/0/1/0 | 1 | 0.0556 |
| **SCoT13** | 1/0/0/1/0/1/1/0/1/0 | 1 | 0.0556 |
| **SCoT13** | 1/0/1/1/1/1/1/1/1/0 | 1 | 0.0556 |
| **SCoT13** | 1/1/0/1/0/1/1/0/1/0 | 1 | 0.0556 |
| **SCoT13** | 1/1/1/1/0/1/1/0/1/0 | 1 | 0.0556 |
| **SCoT28** | 0/0/0/0/0/0/0 | 1 | 0.0556 |
| **SCoT28** | 1/0/0/0/1/0/1 | 1 | 0.0556 |
| **SCoT28** | 1/0/1/0/0/0/0 | 1 | 0.0556 |
| **SCoT28** | 1/0/1/0/0/0/1 | 3 | 0.1667 |
| **SCoT28** | 1/0/1/1/0/0/1 | 9 | 0.5000 |
| **SCoT28** | 1/0/1/1/0/1/1 | 1 | 0.0556 |
| **SCoT28** | 1/1/1/1/0/0/1 | 1 | 0.0556 |
| **SCoT28** | 1/1/1/1/0/1/1 | 1 | 0.0556 |
| **SCoT20** | 0/0/0/0/0/0/0/0 | 1 | 0.0556 |
| **SCoT20** | 0/0/0/0/0/1/0/1 | 1 | 0.0556 |
| **SCoT20** | 0/0/0/0/1/1/0/1 | 1 | 0.0556 |
| **SCoT20** | 0/0/0/0/1/1/1/1 | 1 | 0.0556 |
| **SCoT20** | 0/0/0/1/1/0/0/0 | 1 | 0.0556 |
| **SCoT20** | 0/0/0/1/1/0/1/0 | 1 | 0.0556 |
| **SCoT20** | 0/0/0/1/1/1/0/0 | 1 | 0.0556 |
| **SCoT20** | 0/0/1/1/1/1/1/1 | 1 | 0.0556 |
| **SCoT20** | 0/1/0/1/1/1/1/0 | 1 | 0.0556 |
| **SCoT20** | 0/1/1/1/1/1/0/1 | 1 | 0.0556 |
| **SCoT20** | 1/1/0/1/0/1/1/0 | 1 | 0.0556 |
| **SCoT20** | 1/1/1/1/0/1/0/1 | 2 | 0.1111 |
| **SCoT20** | 1/1/1/1/0/1/1/1 | 3 | 0.1667 |
| **SCoT20** | 1/1/1/1/1/1/0/1 | 2 | 0.1111 |
| **SCoT24** | 0/0/0/1/0/1/1 | 1 | 0.0556 |
| **SCoT24** | 0/0/0/1/1/0/1 | 1 | 0.0556 |
| **SCoT24** | 0/0/0/1/1/1/0 | 1 | 0.0556 |
| **SCoT24** | 0/0/0/1/1/1/1 | 7 | 0.3889 |
| **SCoT24** | 0/0/1/1/0/1/1 | 1 | 0.0556 |
| **SCoT24** | 0/0/1/1/1/1/1 | 3 | 0.1667 |
| **SCoT24** | 1/0/0/1/1/1/1 | 1 | 0.0556 |
| **SCoT24** | 1/1/0/1/1/1/1 | 3 | 0.1667 |
| **SCoT16** | 0/0/1/1/0/1 | 1 | 0.0556 |
| **SCoT16** | 0/0/1/1/1/1 | 1 | 0.0556 |
| **SCoT16** | 1/0/1/0/0/1 | 1 | 0.0556 |
| **SCoT16** | 1/0/1/1/0/1 | 10 | 0.5556 |
| **SCoT16** | 1/0/1/1/1/1 | 2 | 0.1111 |
| **SCoT16** | 1/1/1/1/0/1 | 2 | 0.1111 |
| **SCoT16** | 1/1/1/1/1/1 | 1 | 0.0556 |
